# Prevalence of asymptomatic COVID-19 infection using a seroepidemiological survey

**DOI:** 10.1017/S0950268820002745

**Published:** 2020-11-13

**Authors:** M. Shakiba, M. Nazemipour, A. Heidarzadeh, M. A. Mansournia

**Affiliations:** 1 Cardiovascular Diseases Research Center, Guilan University of Medical Sciences, Rasht, Iran; 2 School of Health, Guilan University of Medical Sciences, Rasht, Iran; 3 Psychosocial Health Research Institute, Iran University of Medical Sciences, Tehran, Iran; 4 Gastrointestinal and Liver Diseases Research Center, Guilan University of Medical Sciences, Rasht, Iran; 5 Department of Epidemiology and Biostatistics, School of Public Health, Tehran University of Medical Sciences, Tehran, Iran

**Keywords:** Asymptomatic infection, SARS-CoV-2, seroprevalence

## Abstract

The prevalence of asymptomatic infection by coronavirus disease 2019 (COVID-19) as a critical measure for effectiveness of mitigation strategy has been reported to be widely varied. In this study, we aimed to determine the prevalence of asymptomatic infection using serosurvey on general population. In a cross-sectional seroprevalence survey in Guilan province, Iran, the specific antibody against COVID-19 in a representative sample was detected using rapid test kits. Among 117 seropositive subjects, prevalence of asymptomatic infection was determined based on the history of symptoms during the preceding 3 months. The design-adjusted prevalence of asymptomatic infection was 57.2% (95% confidence interval (CI) 44–69). The prevalence was significantly lower in subjects with previous contacts to COVID-19 patients (12%, 95% CI 2–49) than others without (69%, 95% CI, 46–86). The lowest prevalence was for painful body symptom (74.4%). This study revealed that more than half of the infected COVID-19 patients had no symptoms. The implications of our findings include the importance of adopting public health measures such as social distancing and inefficiency of contact tracing to interrupt epidemic transmission.

## Introduction

Asymptomatic cases who are carriers without any symptoms introduce a major public health challenge in managing COVID-19 pandemic as they can be an important source of infection that undermines control intervention. Asymptomatic infection is considered as a critical parameter for interventions involving contact tracing, because a similar viral load has been reported among asymptomatic and symptomatic cases [1, 2] and it has been claimed that there is no difference between the transmissibility of asymptomatic and symptomatic cases among close contacts [3]. Furthermore, a previous study suggested that stricter implemented control measures are needed when the infection duration is long or the probability of a susceptible individual to be asymptomatic is large [4]. The reported estimates of asymptomatic COVID-19 infection are widely varied between 6% and 96% [5]. So far, the studies attempting to estimate asymptomatic proportion were based on either SARS-CoV-2 testing from close contacts of confirmed COVID-19 patients and high risk groups [6–12] or a sample of infected persons detected by SARS-CoV-2 test in general population [13, 14]. The former approach may ignore the presence of infection in the general population, and the latter needs to be performed longitudinally to exclude presymptomatic patients. On the contrary, community-based seroepidemiological surveys through detecting specific antibody against SARS-CoV-2 show past infection and can provide better depiction of asymptomatic prevalence and extent of subclinical infection in the population. In this study, we assessed the prevalence of asymptomatic COVID-19 infection (and also separately for each symptom) and associated factors in the whole population using a seroprevalence survey.

## Methods

This study was performed on a subset of participants from a seroprevalence survey in the north of Iran. The details of the study were described elsewhere [15]. Briefly, a random sample of general population permanently residing in Guilan province, irrespective of age, was selected through stratified multistage (cluster) sampling and during 11–19 April 2020. Residents of institutional living centres such as nursing homes, prisons and boarding schools, and those people who did not want to participate, were under active treatment of COVID-19, or had any contraindication to venepuncture were not invited to the study. On the day of attendance at health care centre, an electronic questionnaire adopted from the WHO [16] including demographic information, comorbidities and series of COVID-19 symptoms that had been present during the preceding 3 months was completed for each participant. Then, a 50 μl of capillary blood was taken from the participants and tested for SARS-CoV-2 specific antibody using VivaDiag COVID-19 IgM/IgG rapid test (VivaChek). The sensitivity of the test was reported to be 83.3% for IgG and both IgM and IgG, and 83.3% for IgM and either IgM or IgG [17]. Of 528 valid blood test results, 117 seropositive subjects were included in this study. Asymptomatic infection was defined as having no symptoms associated with COVID-19 including fever, dry cough, tiredness, chill, sore throat, ache and pain, runny nose, shortness of breath, headache, vomiting, diarrhoea and wheezing. Symptom-specific asymptomatic infection was defined as not developing that symptom. The prevalence of asymptomatic COVID-19 infection and symptom-specific asymptomatic infection with 95% confidence intervals (CIs) were estimated considering the multistage clustering design of the sampling. Specifically, an inverse probability weighting with weights equal to the inverse of the probability of selection was used to adjust for selection bias because the probability of selection varied over the participants [18]. Cluster robust standard errors were used to account for clustering [19].

## Results

A total of 65 out of 117 seropositive subjects reported no symptoms associated with COVID-19 in 3 months preceding the study. The design-adjusted prevalence of asymptomatic infection was 57.2% (95% CI 44–69). Twenty-two subjects had a history of contacts to confirmed COVID-19 patients. The prevalence of asymptomatic infection in subjects with previous contacts to COVID-19 cases (12%, 95% CI 2–49) was significantly lower than others without previous contacts (69%, 95% CI 46–86). The odds of symptomatic infection in contacts of COVID-19 cases was 17.2 (95% CI 4.3–68.1) times of non-COVID-19 contacts. The resulting odds ratio adjusted for sex, age, place of residence, job, education level, obesity and having comorbidities was 45.9 (95% CI 10.3–206). There was no strong evidence for the association between asymptomatic infection with sex (*P*-value = 0.20), age group (*P*-value = 0.69), job (*P*-value = 0.41), place of residence (urban or rural) (*P*-value = 0.64), educational level (*P*-value = 0.95), obesity (*P*-value = 0.31) or having any comorbidities (*P*-value = 0.25). Of the 117 subjects whose test was positive, 10 (8.5%) reported typical symptoms, and 44 (38%) reported atypical symptoms. The prevalence estimates of asymptomatic infection with 95% CIs based on COVID-19-like symptoms and combination of typical criteria for COVID-19 used by the WHO (i.e. fever, dry cough and tiredness) are provided in Table 1. The lowest proportion was for painful body (74.4%) that was the most prevalent symptom in seropositive subjects.

**Table 1.** The prevalence of symptom-specific asymptomatic COVID-19 infection based on symptoms

Symptom(s)	Prevalence of asymptomatic infection (%)	95% CI
Fever	78.5	44.2–94.4
Cough	79.6	57.8–91.7
Sore throat	81.6	52.5–94.7
Tiredness	76.5	50.2–91.3
Painful body	74.4	42.3–92.0
Runny nose	91.1	63.9–98.3
Dyspnoea	89.5	36.1–99.2
Headache	83.8	50.2–96.3
Diarrhoea	94.2	89.1–96.9
Fever + cough + tiredness	87.6	46.7–98.3

## Discussion

In this study based on a seroprevalence survey, the prevalence of asymptomatic infection of SARS-CoV-2 was 57%, indicating that more than half of the infected patients had no symptoms. Asymptomatic prevalence in previous studies using SARS-CoV-2 testing on general population was estimated to be 43–45% [13, 14]. The prevalence was conservatively estimated to be 30% after accounting for presymptomatic cases [5]. The main difference between this study and previous reports is that the current findings were based on serosurvey among a representative sample of general population. However, the sample was not large enough to detect associations between asymptomatic prevalence with underlying variables. Our study showed that the asymptomatic rate was highly dependent on previous contact to COVID-19 cases, and was significantly lower in the contacts of COVID-19 patients compared to non-previous contacts (12% *vs.* 69%). This is in line with the overall report of 15% for prevalence of asymptomatic infection by a previous meta-analysis conducted on studies exploring close contacts of COVID-19 patients [20] in which about 10–90% of primary asymptomatic persons developed symptoms of COVID-19 after 2–14 days of follow-up [6, 8, 21].

Because of evidence revealing transmission of SARS-CoV-2 from asymptomatic cases to others [14, 22], the high prevalence of asymptomatic infection gleaned from this study highlights the importance of social distancing and other protective measures such as wearing face masks in the community. The finding also questioned the efficacy and relying on contact tracing as the only approach for containment of COVID-19 and indicated that only testing those with symptoms may not interrupt epidemic transmission. A previous study showed that 10–15% of cases are expected to generate at least one unidentified secondary case which would need detecting by other means [23].

This study had the advantage of including a wide range of age groups from less than 5 years old to more than 80 years. Despite the lack of significant findings, current study revealed two asymptomatic cases out of four seropositive children less than 5 years and 13 out of 19 people older than 60 years. The evidence about the asymptomatic prevalence in children is very scarce [6] and the findings of current study may shed light on the fact that all age groups are at risk of getting SARS-CoV-2 infection and yet to be asymptomatic.

In current study, since the active COVID-19 cases were excluded from the original research, we adjusted the prevalence using the total hospitalised cases in the study period. The difference in the estimated prevalence was very trivial, changing from 57.2% to 57.4%.

This study had some limitations. Recall bias for minor symptoms is likely. However, recall bias was very likely if symptoms were asked for in a period longer than the previous 3 months. Since the study was conducted right after the peak of the epidemic wave, recall bias for underestimating moderate and severe symptoms was less likely. On the contrary, the overlap of influenza season with the peak of the epidemic wave may result in an overreporting of symptoms that are similar between the two diseases. Second, due to limited sample sizes for some combinations of predictors and asymptomatic infection, the confidence limits were unrealistically large suggesting sparse-data bias [24–26]. Finally, imperfect rapid test accuracy in terms of test sensitivity may result in false negatives. We did not adjust for diagnostic accuracy of the test, though specificity was perfect and there is no evidence of association between symptoms and becoming false negative so no adjustment is needed.

## Conclusion

This study revealed a high prevalence of asymptomatic infection among seropositive SARS-CoV-2 patients. The asymptomatic prevalence was highly dependent to previous contact to COVID-19 patients. Future studies are needed to clearly elucidate the transmissibility of asymptomatic cases. Yet, because of current evidence of asymptomatic transmission of COVID-19, the current study findings reinforces that adopting control measures such as social distancing and wearing face masks in the community is very critical for managing the epidemic.

## Data Availability

The data that support the findings of this study are available from the Guilan University of Medical Sciences. Restrictions apply to the availability of these data, which were used under licence for this study. Data are available from the authors with the permission from the Guilan University of Medical Sciences.
